# Non-Traumatic Laryngeal Fractures: A Systematic Review

**DOI:** 10.4274/tao.2020.6093

**Published:** 2021-03-26

**Authors:** Noor Khalid, Muhammad Bilal, Muhammad Umer

**Affiliations:** 1 Department of Otorhinolaryngology, Rawalpindi Medical University, Rawalpindi, Pakistan; 2 Department of Otorhinolaryngology, Foundation University Medical College, Islamabad, Pakistan; 3 Department of Otorhinolaryngology, Army Medical College, Rawalpindi, Pakistan

**Keywords:** Larynx, spontaneous fractures, thyroid cartilage, management, computed tomography, systematic review

## Abstract

Non-traumatic laryngeal fractures are an extremely uncommon presentation, and the diagnosis can be missed. Recognizing these fractures is important to appropriately direct management because most have a good prognosis and result in complete recovery. This article aimed to review the characteristics of all documented cases of non-traumatic fractures of the larynx. We sought to address questions related to the etiology, clinical presentation, and diagnostic assessment of this condition and provide recommendations about the management of these fractures. Electronic databases, mainly PubMed and Google Scholar, were searched for relevant literature with no language or time restrictions. Since 1950, 15 cases of non-traumatic laryngeal fractures have been documented in the medical literature. Out of these, thyroid cartilage fractures have been described in 14 patients, while only one instance demonstrated a fracture in the cricoid cartilage. Patients were managed conservatively using voice rest and observation with complete recovery in all cases. All patients who present with odynophagia, hoarseness, and tenderness over the thyroid cartilage after an episode of severe coughing or sneezing, should be evaluated for a thyroid cartilage fracture using laryngoscopy and computed tomography scan. Management of the airway should be the primary priority in any laryngeal injury, and further management performed after the airway is stable.

## Introduction

Non-traumatic fractures of the larynx remain a less understood phenomenon in the medical literature. Since the first case of a spontaneous thyroid cartilage fracture documented in 1950, our knowledge regarding the etiology and the pathophysiology of these fractures has not substantially improved (1). There are yet no established guidelines for the management of these cases. Fractures of the laryngeal cartilages without a prior history of trauma are a rarely encountered entity; however, it is vital to be able to recognize this pattern of injury, as most of these fractures have an excellent prognosis without the need for surgical intervention.

This systematic review summarizes and critically analyzes the available literature, which includes 15 cases documented from 1950 through 2020 (1, 2, 3, 4, 5, 6, 7, 8, 9, 10, 11, 12, 13, 14).

## Methods

### Review Protocol

The protocol of our review follows the Preferred Reporting Items for Systematic Reviews and Meta-Analyses Protocol (The PRISMA Statement) guidelines (15). The protocol was previously not registered.

### Search Strategy and Study Selection

We conducted a systematic literature review using Harzing’s Publish or Perish software (16). Two authors (M.B. and M.U.) independently searched PubMed Central and Google Scholar databases for relevant articles up to October 2020. Other resources used were Cureus and ResearchGate. A broad core search term “laryngeal fracture” was used. Articles dealing with traumatic fractures and those irrelevant to our study were excluded. We included case reports or case series documenting non-traumatic laryngeal fractures, with no language or time restrictions. The references of the included articles were also used to identify additional cases. We screened all case reports manually for duplication and relevance, and any uncertainty encountered during the process was resolved by mutual consensus.

### Quality Assessment of Included Case Reports

We assessed the quality of the included case reports by comparison with the 2013 Case Report guidelines (CARE checklist) (17) and the Joanna Briggs Institute (JBI) Critical Appraisal Checklist for Case Reports (18). An evaluation form was used to score each article against the checklist items. Each positive item on the form conferred a score of one. In this manner, the articles were scored out of 28 for the CARE checklist, and 8 for the JBI checklist. To minimize bias, two reviewers independently assessed each case report, and the mean of the two scores was then calculated.

### Data Retrieval and Analysis

After thorough research and screening, 14 articles that documented 15 cases were selected for review. Data retrieved from case reports included the title of study, name(s) of the author(s), year of publication, age and sex of patients, fracture type, etiology, precipitating event, comorbid conditions, signs and symptoms, investigations, and management plan. Data were then recorded in Microsoft Excel 2016 (Microsoft Corporation, Redmond, Washington, USA) and analyzed.

## Results

**Figure 1:**
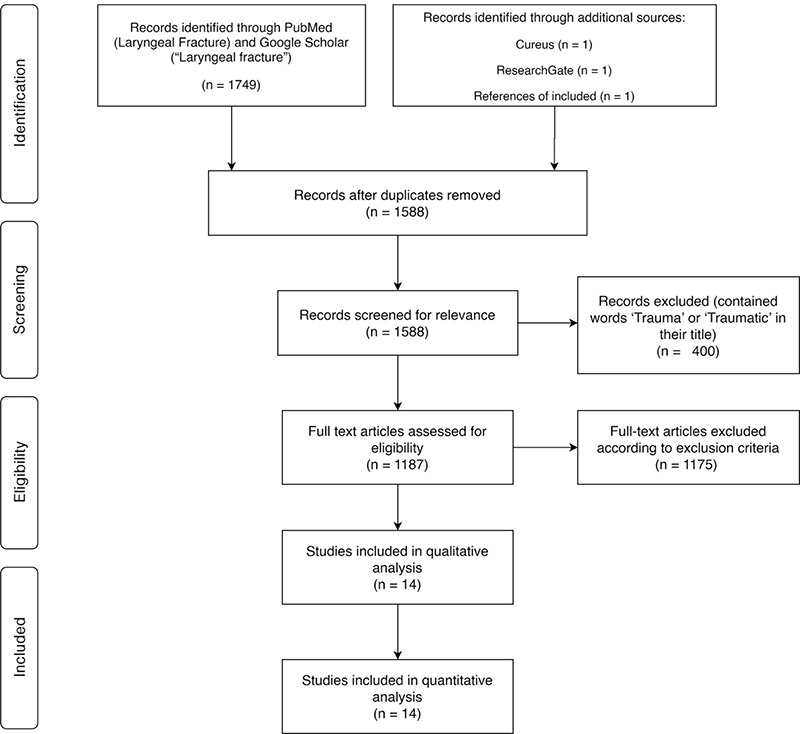
PRISMA Diagram of the literature retrieval process n: Number

The initial PubMed search for “Laryngeal Fracture” revealed 828 results, while a Google Scholar search revealed 920 results. Three articles were identified from additional sources. After removing duplicates and excluding irrelevant studies, 14 articles were selected for analysis. The PRISMA flowchart for the literature review process is shown in Figure 1.

Until now, 15 cases of spontaneous laryngeal fractures have been documented, 14 of which reported thyroid fractures, while one study reported a fracture of the cricoid cartilage. For the sake of simplicity in analysis, all fractures were assigned a grade using the Schaefer Fuhrman’s classification of laryngeal injuries (19). Mildly displaced fractures were classified as grade II alongside nondisplaced fractures. Table 1 provides a summary of the characteristics of all included case reports.

The included cases were further analyzed based on their presentations and management, as shown in Table 2. Only the detailed characteristics of thyroid fractures (1, 2, 3, 4, 5, 6, 7, 8, 9, 10, 11, 12, 13) were analyzed, and the case that demonstrated cricoid cartilage fracture (14) was excluded from the analysis. We did so because no other cases of spontaneous cricoid cartilage fractures have been reported in the literature, and hence, we did not have any study to compare it.

### Pathophysiology

Non-traumatic fractures of the larynx occur when the pressure inside the laryngotracheal complex rises in the presence of a closed glottis. This phenomenon occurs during coughing and swallowing, and results in a substantial force being exerted on the cartilages of the larynx. Amongst the documented cases, sneezing was the most common cause of thyroid fractures (n=9, 64.3%), followed by coughing (n=3, 21.4%) and swallowing (n=2, 14.2%). Sneezing on a closed airway increases the pressure by 38 times, which results in a higher risk (3). The precipitating event of the only reported non-traumatic cricoid fracture was unclear (14).

### Patient Characteristics

Fractures of the thyroid cartilage made up 93.3% (n=14) of all non-traumatic laryngeal fractures. These fractures occurred exclusively in males (n=14, 100%) with the mean age of patients being 40 years old (range: 29-61). Most patients (n=9, 64.3%) were otherwise perfectly healthy, with no known comorbid conditions. Of the remaining, the most common comorbid condition was smoking, which was observed in three patients (n=3, 21.4%). Other reported conditions include gastrointestinal reflux disease (GERD), asthma, type 1 diabetes mellitus (DM), hypertension, and obstructive sleep apnea, all of which had a frequency of one.

Only one case report reported the ethnicity of the patient to be African-American (12). The rest of the studies do not give any information regarding the patient’s ethnic background. Other patient characteristics, including occupation and geographical residence, were not reported for any case.

On the other hand, there has only been one case of a cricoid fracture documented, and the patient was a 69-year-old female with significant comorbidity. She had a prolonged history of DM and was on dialysis due to end-stage renal disease. Her ethnicity and occupation were not reported (14).

### Fracture Characteristics

Almost all thyroid fractures occurred in the anterior part of the thyroid cartilage. Most fractures occurred in the paramedian plane (n=10, 83.3%). Soft tissue infection with the fracture was present only in two (14.3%) cases (8, 11). Most fractures belonged to Shaefer grade II (n=12, 85.7%) with slight or no displacement of the fracture segments. Only one case reported significant displacement of the fracture fragments, which was due to compression by an anterior neck abscess (11). The case with the cricoid cartilage fracture demonstrated a posterior lamina fracture with airway compromise but no soft tissue infection (14).

### Clinical Presentation

The main presenting complaints included odynophagia (n=12, 85.7%), hoarseness (n=13, 92.9%) and tenderness to palpation over the thyroid cartilage (n=9, 64.3%). 

Fenig et al. (5) suggested that the triad of odynophagia, hoarseness, and dysphagia after an episode of coughing or sneezing should raise the suspicion of thyroid fracture. However, analysis of the included cases showed that this triad was documented only in four cases (28.6%). Alternatively, the triad of odynophagia, dysphonia, and tenderness was present in nine of 14 cases (64.3%). Crepitus was present only in five cases (35.7%) although it is a strong indicator of laryngeal injury. The only reported symptom of a non-traumatic cricoid fracture was intermittent dyspnea (14). Further cases need to be documented for more information regarding the presentation of these fractures.

### Assessment and Diagnosis

The two primary investigations in the diagnosis of this condition are the examination of the larynx by endoscopy and computed tomography (CT). Of all reported cases, 85.7% patients underwent endoscopic examination via flexible nasendoscopy, laryngoscopy, or bronchoscopy. The most frequently encountered findings were edema over the laryngeal inlet or in the subglottic region (n=9, 75%) and hematoma over the ipsilateral vocal cord (n=6, 50%). All cases showed normal vocal cord movement (n=10, 100%). This investigation is important for its value in the assessment of the airway status and in ruling out other (and more common) causes of hoarseness including laryngitis, benign vocal cord lesions, and vocal cord paralysis.

CT is the most significant diagnostic investigation. A CT scan of the neck outlined the fracture in almost all of the cases. All cases underwent CT imaging except the case reported in 1950 (1), which can be attributed to the nonavailability of CT scans during that period. For that patient, the diagnosis was made by physical examination, which showed a fracture line and slight mobility of fracture segments. A plain radiograph performed was not diagnostic (1). Similar principles were used for the diagnosis of cricoid fracture. Laryngoscopy and bronchoscopy were performed, followed by CT scan which revealed the fracture (14).

### Management

All patients who had thyroid fractures were managed conservatively (n=14, 100%) with complete recovery after a median follow-up time of 8.5 days. No fracture required surgical fixation or compromised the airway to warrant a tracheostomy. The management protocol was not stated in detail for most case reports; however, most patients were managed using voice rest (n=9, 64.3%) and observation.

**Figure 2:**
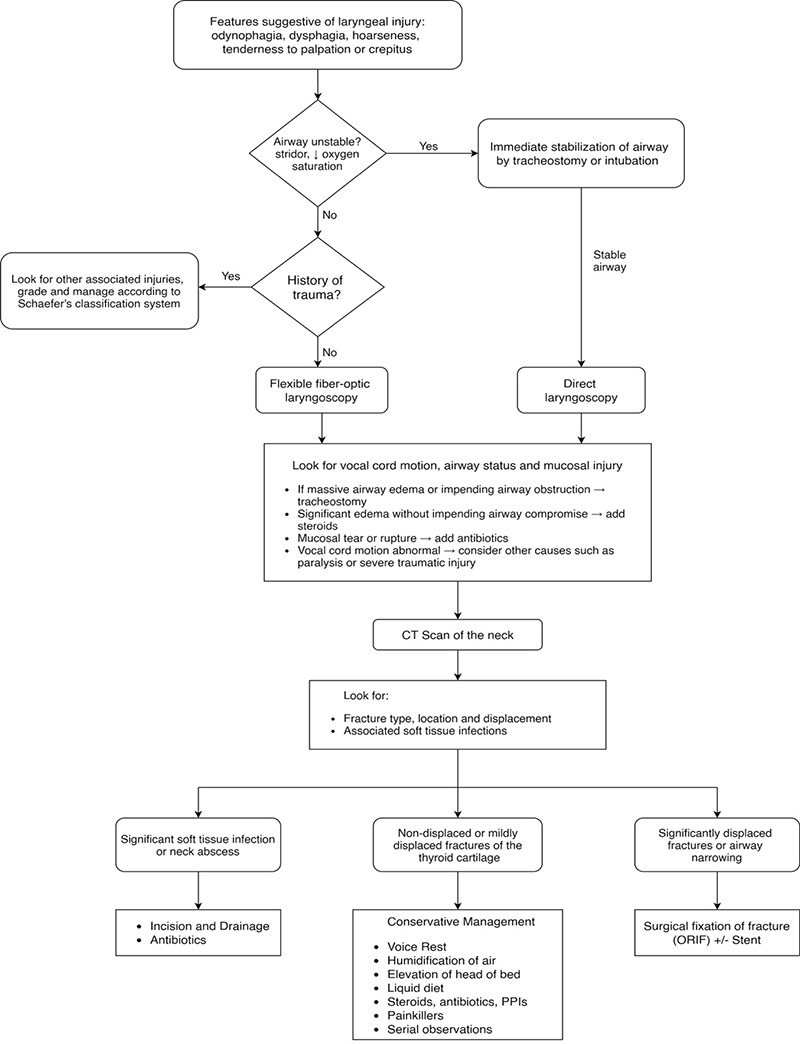
Algorithm summarizing the management principles of all included cases of thyroid fractures CT: Computed tomography, ORIF: Open reduction and internal fixation, PPI: Proton pump inhibitor

Analysis of the cases shows that steroids were administered in seven cases (n=7, 50%) whereas one case reported the use of nonsteroidal anti-inflammatory drugs (NSAIDs), resulting in a similar outcome (8). Alexander and Toynton (4) suggested that antibiotics should be used only in cases where there is mucosal injury. Of all included cases, five cases received antibiotics (35.7%), however the precise antibiotic used and the details of its administration were lacking. Many reports have also mentioned use of soft and liquid diet in the treatment regime (1, 2, 5, 11). Based on observations from the documented cases, the main principles for managing non-traumatic thyroid cartilage fractures are summarized in Figure 2.

Alternatively, the cricoid fracture compromised the airway causing intermittent dyspnea, so a tracheostomy was performed (14). Ideally, the fracture should have been surgically repaired; however, the patient was not fit for surgery. The patient was managed conservatively by airway preservation and observation.

## Discussion

This article is the first comprehensive review summarizing the characteristics of laryngeal fractures that occur without a prior history of trauma. ​While most of these fractures occur in middle-aged men, their presentation and examination findings vary from patient to patient. After diagnosis by CT and laryngoscopy, these fractures can be successfully managed conservatively.

The pathophysiology of these fractures has been described in detail by many case reports (3, 5, 9) and is satisfactorily explained by the biomechanics of coughing and sneezing (20, 21). Increased intraluminal pressure during these reflex processes has been described as the causative event, however, it is still unclear as to why certain individuals are more susceptible to this injury than the others. Quinlan (1) suggested the possibility of a congenital defect in the cartilage which predisposes to these fractures, while a few authors have suggested that inflammatory or neoplastic processes result in pathological fractures (11). Most authors, however, believe defects in ossification and mineralization of the thyroid cartilage to be the primary culprit (4, 7). This hypothesis is favored because the ossification process differs between the two sexes; and therefore, it may explain the predominance of laryngeal fractures in men (22, 23, 24).

A problem with this etiological hypothesis is that it does not explain why the only documented case of a cricoid fracture involved a female patient (14). Cricoid cartilage is thicker and stronger than the thyroid cartilage, and it ossifies later (25, 26). The case involved a 69-year-old lady with a posterior cricoid fracture. Since the patient did not recall any precipitating event and no other similar case has been reported in the literature, it is possible that her fracture occurred secondary to blunt trauma to the neck, which the patient did not recall at the time of presentation. This is a common phenomenon in older adults, and studies show that many elderly patients do not remember injuries well (27). Besides, abuse is often not reported by elderly patients (28). Additional cases need to be documented before any conclusions can be drawn regarding cricoid fractures.

Most non-traumatic fractures of the larynx belonged to Schaefer grade II. The Schaefer classification of traumatic laryngeal fractures and the management plan for each fracture type is summarized in Table 3. Faden et al. (3) highlighted that the management of grade II traumatic injuries often requires a tracheostomy (19). On the other hand, non-traumatic grade II injuries were all managed conservatively without the need of a tracheostomy. This difference in treatment has been attributed to the mechanism of injury, and it further emphasizes that the mechanism of injury must be considered during its management. Additionally, traumatic laryngeal injuries are often associated with other injuries, while non-traumatic fractures are almost always an isolated phenomenon and hence can be managed successfully with a less aggressive approach (29). A separate classification system should thus be developed for the grading and management of non-traumatic laryngeal fractures.

A controversy exists over the use of steroids in laryngeal trauma. Traditionally, steroids have been administered to all patients with edema of the larynx and were believed to relieve laryngeal edema and its exacerbation after intubation and bronchoscopy (30). However, recent guidelines on the use of steroids in these injuries are unclear (31, 32, 33). Fifty percent of the reviewed cases mentioned steroids to be a part of their treatment regimen. For the other half, steroids were either not administered or their use was not documented. Of note, all patients reported similar outcomes, i.e., complete recovery. In the absence of established guidelines, a reasonable approach would be to use steroids in cases with significant airway edema only. Further studies are needed before any objective recommendations can be made.

In addition, there are no clear principles about the use of antibiotics in these patients. Based on observations from the reviewed cases and recommendation by Alexander and Toynton (4), antibiotics should be administered only if there is associated soft tissue infection or if laryngeal mucosa is injured. We identified two cases where antibiotics were administered without any of these indications (3, 5). It is possible that antibiotics were added to the treatment regimen as a “general practice,” keeping in view the universal tendency of doctors and hospitals to overuse these drugs (34, 35). However, since two cases of spontaneous laryngeal fractures did have an associated infection necessitating their use, it is unclear whether antibiotics should be administered as routine prophylaxis to prevent infection or not. Further research is required to answer this question.

An important observation made during the quality assessment of the case reports was the fact that no case report completely conformed to the principles stated in quality assessment guidelines. This finding is similar to the overall low quality of published case reports as highlighted by many studies (36, 37).

Out of 15 cases of non-traumatic laryngeal fractures reported, 14 cases did not report the ethnicity or racial origin of the patient. Many medical conditions have specific ethnicity patterns, so this information should not be regarded as insignificant (38, 39). Furthermore, most case reports do not provide sufficient detail about their treatment regimen, mentioning it only as “conservative.” Details regarding the dosage and administration of drugs have been excluded. All future authors need to be thorough in their documentation since no treatment guidelines exist for this condition, and case reports are the only available source of evidence.

This review has several limitations. Primarily, there is a lack of documented cases, with extensive literature review revealing only 15 instances of such fractures. Second, as described above, the included case reports are lacking in important information regarding patient characteristics and management protocol, and hence no concrete recommendations can be made. Third, case reports are the lowest level of evidence, and further studies are needed before evidence-based guidelines can be constructed for clinicians to be able to manage this condition effectively (40). This review is the first attempt to organize all the published knowledge about this entity, and we hope that it will pave way for further research on this topic.

## Conclusion

Non-traumatic fractures of the larynx are rare clinical entities. Although important information regarding the presentation and management of these fractures can be obtained by reviewing cases documented in the literature, areas of further research remain. All such cases encountered in clinical practice must be documented in the form of high-quality case reports for evidence-based guidelines to be constructed.

**Main Points**

• Fractures of the larynx can occur without a history of trauma and have been reported in the past after sneezing or coughing.

• These patients present with symptoms of odynophagia and hoarseness. Neck palpation may indicate tenderness and crepitus.

• The diagnosis is made on CT scan and endoscopic examination. Fractures occur mostly in the anterior part and are non-displaced or mildly displaced.

• Patients are managed conservatively. The decision to administer steroids or antibiotics should be based on the patient’s clinical status and the physician’s preference.

## Figures and Tables

**Table 1:**
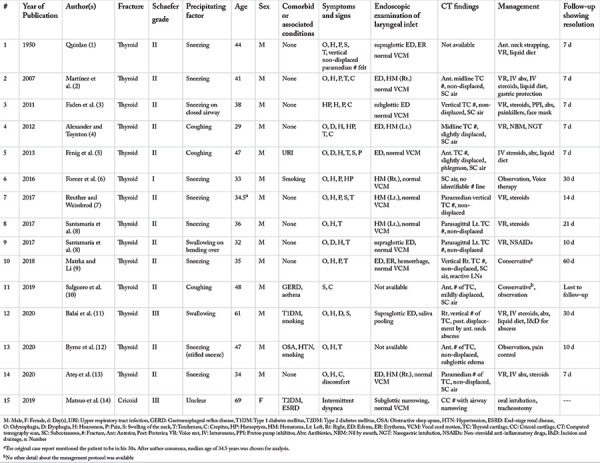
Characteristics of the included studies demonstrating non-traumatic fractures of the larynx (n=15)

**Table 2:**
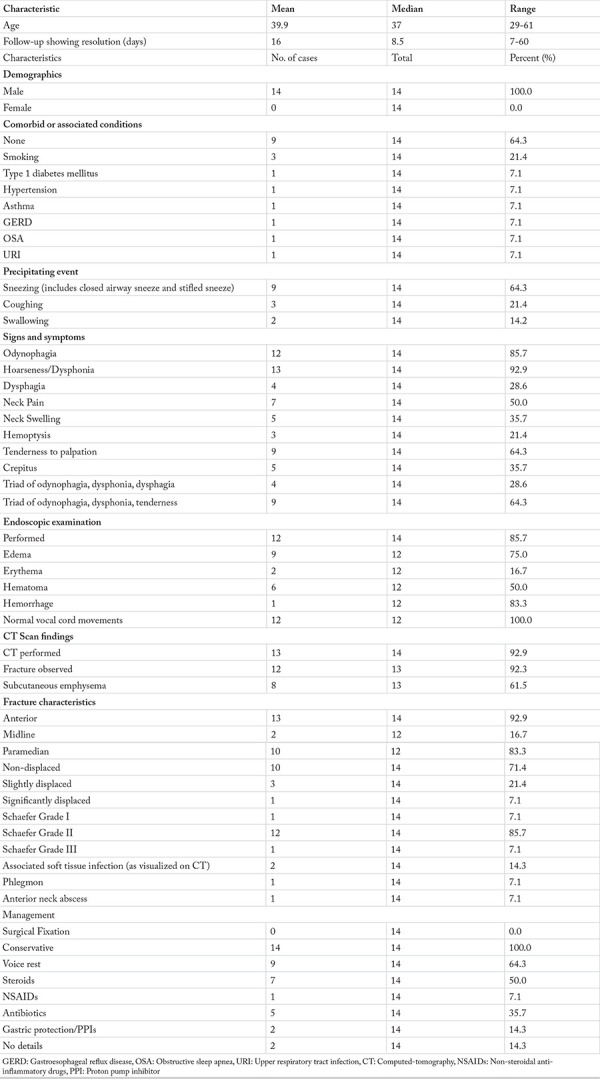
Analysis of selected characteristics of non-traumatic thyroid fractures (n=14)

**Table 3:**
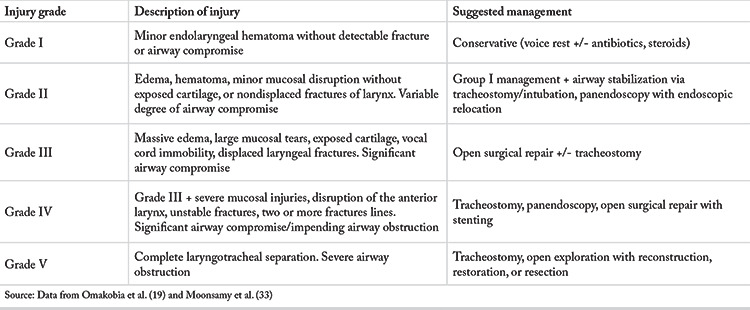
Schaefer Fuhrman’s Classification of traumatic laryngeal injuries and suggested management according to injury grade
